# Analysis of the effect of joint roughness on the failure mechanism of jointed rock mass under direct shear loading

**DOI:** 10.1371/journal.pone.0310893

**Published:** 2025-01-24

**Authors:** Zhongxing Wang, Yuanming Liu

**Affiliations:** School of Civil Engineering, Guizhou University, Guiyang, Guizhou, China; Henan Polytechnic University, CHINA

## Abstract

The mechanical properties of jointed rock bodies are important in guiding engineering design and construction. Using the particle flow software PFC2D, we conducted direct shear test simulations on joints with various inclinations and five different roughness levels to examine the models’ crack extension penetration paths, damage modes, and strength characteristics. The findings indicate that the direction of the joint influences the pattern of the rock crack and its penetration route. Under forward shear, the rock bridge creates a notched through surface, whereas under reverse shear it creates two adjacent through surfaces, categorised into four types of crack consolidation between joints with different inclinations: ‘end to end’, ‘the end is connected to the middle’, ‘end connection’, ‘first outward expansion and then rock bridge destruction’. Variations in joint inclination and roughness can alter the mechanical properties and damage patterns of joint specimens. The ‘climbing’ and ‘gnawing’ effects determine the peak shear strength of the rock body at the joint section. It is vital to consider these factors when assessing the joint’s characteristics. The damage effect is determined by the joint inclination and joint roughness. When the main damage effect changes from ‘creeping’ slip to ‘gnawing’ damage, increasing joint roughness enhances the shear strength. Nevertheless, under the same ‘gnawing’ damage effect, augmenting joint roughness weakens the mechanical properties of the rock bridge, and as roughness increases, the shear strength decreases. For example, at a joint inclination of 30°, the shear strength increases by 20.1% as the JRC (Joint Roughness Coefficient) increases from 0 to 5. At a joint inclination of 60°, the shear strength decreases by 10.7% as the JRC increases from 0 to 10.

## 1. Introduction

A multitude of non-penetrating jointed rock structures are present in practical engineering. Owing to the presence of joints, their mechanical properties are more intricate than those of undamaged rocks [1, 2]. Destruction of engineering rock formations mainly involves the destruction of joints and rock bridges in tandem. The cracking and penetration damage patterns within crackd rock bodies, along with joint interaction, significantly affect the rock body’s strength characteristics [3, 4]. Researchers from both domestic and international institutions have explored non-through joints using various methods, including theoretical derivation, field testing, model testing, and numerical simulations, resulting in multiple findings [5–9].

It has been discovered that many engineering events, including slope instability and tunnel collapse harm, are directly linked to changes in joint morphology, the strength features of joint surfaces, and stress conditions within the rock mass [10, 11]. Therefore, it is of great significance to conduct an in-depth study on the joints of non-through jointed rock bodies [12–14]. The investigation into the influence of joint inclination and roughness on the mechanical properties and damage modes of rock bodies with non-through joints has the potential to establish a theoretical framework for accurately assessing the quality of rock formations, thereby ensuring safe construction practices and reducing project costs. This holds significant importance for the practical application of the project [15, 16].

Lajtai established the strength theory of non-through joints, which is now widely used. The damage to rock bridges is classified into three modes: tensile, shear, and extrusion damage[17–19]. Lajtai’s theory is considered a groundbreaking contribution in this field. Jennings put forth the Jennings method to examine the concept of extensive penetration of non-penetrating jointed rock formations. Xia et al. [20] prepared two types of specimens with different joint roughness as well as different distribution of joints and rock bridges, respectively, and established a model for the weakening of rock bridges in non-through joints based on the test results. Liu et al. [21] conducted direct shear tests on three types of non-through joints containing toothed joints, and investigated the mechanism of weakening mechanical properties of rock bridges in non-through joints and the penetration model. Liu et al. discussed the effects of joint roughness and joint spacing on the shear strength of multi-jointed rocks by carrying out direct shear tests on rock-like rocks containing double and triple joints. Wong et al. [22] conducted direct shear modelling tests on different rock masses such as marble and granite to investigate the effect of rock mass properties on penetration patterns. In terms of numerical simulation, Liu et al. [23] used discrete element software PFC2D for simulation on the basis of relevant model tests to study the mechanical properties of normal stress and connectivity on non-penetrating joints rock mass under direct shear conditions. Chen et al. [24] used experiments and numerical simulations to analyse the influence law of joint roughness on the shear strength of non-through joint rock mass.

Currently, physical model tests and numerical simulations of the shear mechanical properties of non-through joints are primarily based on various characteristics such as the number of joint bars, joint distribution location, joint inclination, and connectivity [25–27]. However, there is comparatively limited research on the shear mechanical properties of joints with differing roughness in anisotropic joints. Therefore, this paper establishes a numerical model by PFC2D to analyse the shear strength as well as the damage mode of joints under different inclination angles and different joint roughness, so as to reveal the shear damage process of joints in a more comprehensive and more graphic way.

## 2. Numerical modelling

### 2.1 Modelling process

**Fig 1:**
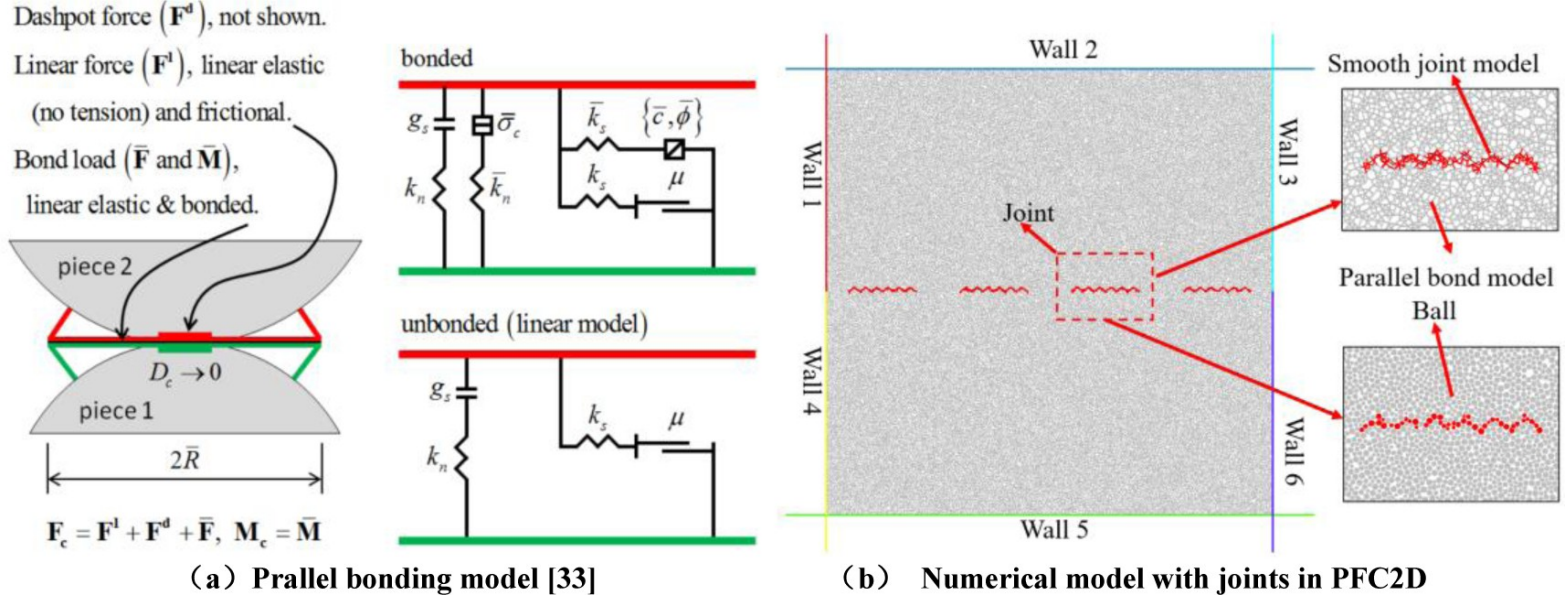
Illustration of the parallel bonding model and Numerical model with joints in PFC2D. (a) Prallel bonding model [33]. (b) Numerical model with joints in PFC2D.

A substantial body of research has demonstrated the efficacy of PFC as a reliable tool for modelling rock damage, with particular emphasis on the modelling of crack propagation processes, including crack processes at the microscopic level. This research has revealed a number of advantages associated with the use of PFC in this context. In this paper, PFC 2D is employed to construct a simulation model comprising a variety of models constructed in the software, including linear, contact bonding and parallel bonding models. According to the relevant research results of scholars at home and abroad, it is confirmed that the Parallel Bond Model (PBM) is more suitable for simulating the mechanical properties and damage modes of rock-like materials [28–32]. The parallel bonding model enables the transfer of forces and moments between neighbouring elements, a property that allows the model to characterise the ontological relationships between cementitious materials at the particle level, as illustrated in Fig 1(A). The parallel bonding model is selected for simulation purposes as it produces more consistent results with the actual situation.

The PFC software generates three main types of prefabricated joints for the simulation of jointed rock bodies: the particle deletion method, the smooth joint model, and the assignment method. In order to investigate the effect of joint roughness, a smooth joint model is employed to generate joints, with four joints generated per numerical model. The initial step in simulating rock materials is to determine the size of the grains. An excessively large particle size will result in a distorted model that will not accurately reflect the reality of the damage. A particle size that is too small will result in an increased computational burden and a correspondingly longer computation time. The final particle size is set to 0.087–0.144mm, with a particle porosity of 0.02. Six walls are defined to form a rectangular range of 200mm×200mm. The shear box is replaced with the wall defined in the procedure, with the 1#, 2#, 3# walls forming the upper shear box and the 4#, 5#, 6# walls forming the lower shear box. The shear loading process is initiated at a rate of 0. A horizontal movement of 0.06mm/s to the right is applied to the 4#, 5#, and 6# walls, which comprise the lower shear box. The 2# and 5# walls are kept immobile by controlling the displacement servo between them. The normal stress for the preset value is 1 MPa, and finally, the desired joints are imported into the model. The numerical model containing the joints is depicted in Fig 1(B).

Barton and Choubey initially delineated ten typical profiles, each exhibiting distinct joint roughness, corresponding to JRC values ranging from 0 to 20 [34]. However, since the JRC value determined in this way is largely dependent on human visual inspection, it is inevitable that errors will occur. Xie et al. [35] subsequently enhanced the traditional Koch curve, devising an estimation formula that delineates the correlation between the JRC value and the fractal dimension through the theoretical fractal model of the established joint profile. This formula permits the calculation of the degree of joint roughness.


$$JRC=85.267{\left(D-1\right)}^{0.5679}$$ (1)

D=lg4/lg{2[1+cos(arctan(2h/L))]} (2)

The average basal length, L, and the average height of the joint roughness, are the variables in question.

**Fig 2:**
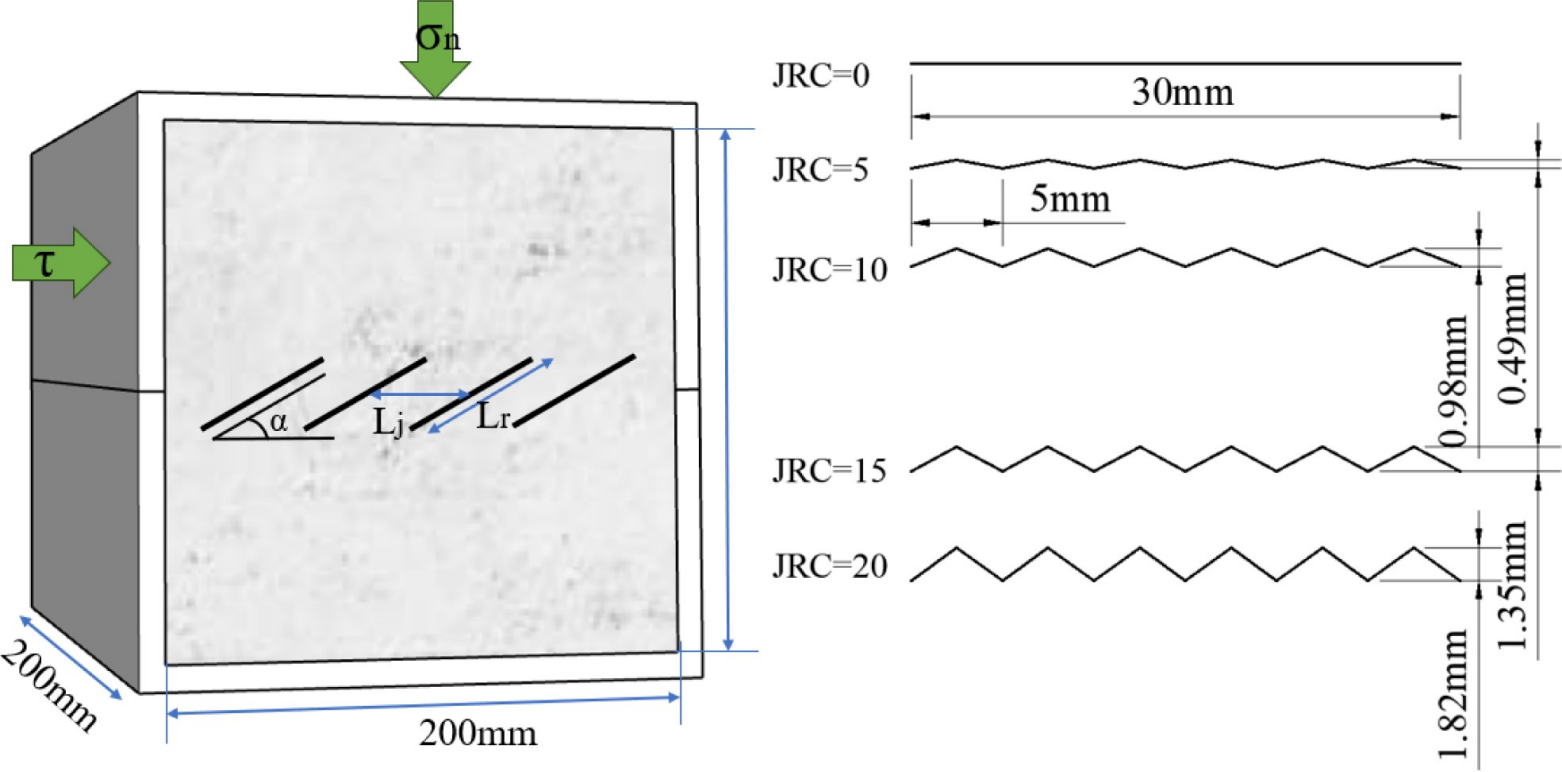
Schematic diagram of different roughness joints as well as loading.

In this paper, the effect of joint roughness on shear strength is investigated by selecting five cases of JRC = 0, 5, 10, 15 and 20. The geometry of the specific joints is derived according to the JRC estimation formula, as shown in Fig 2.

### 2.2 Verification of the reliability of the numerical model with regard to the fine-scale mechanical parameters

**Fig 3:**
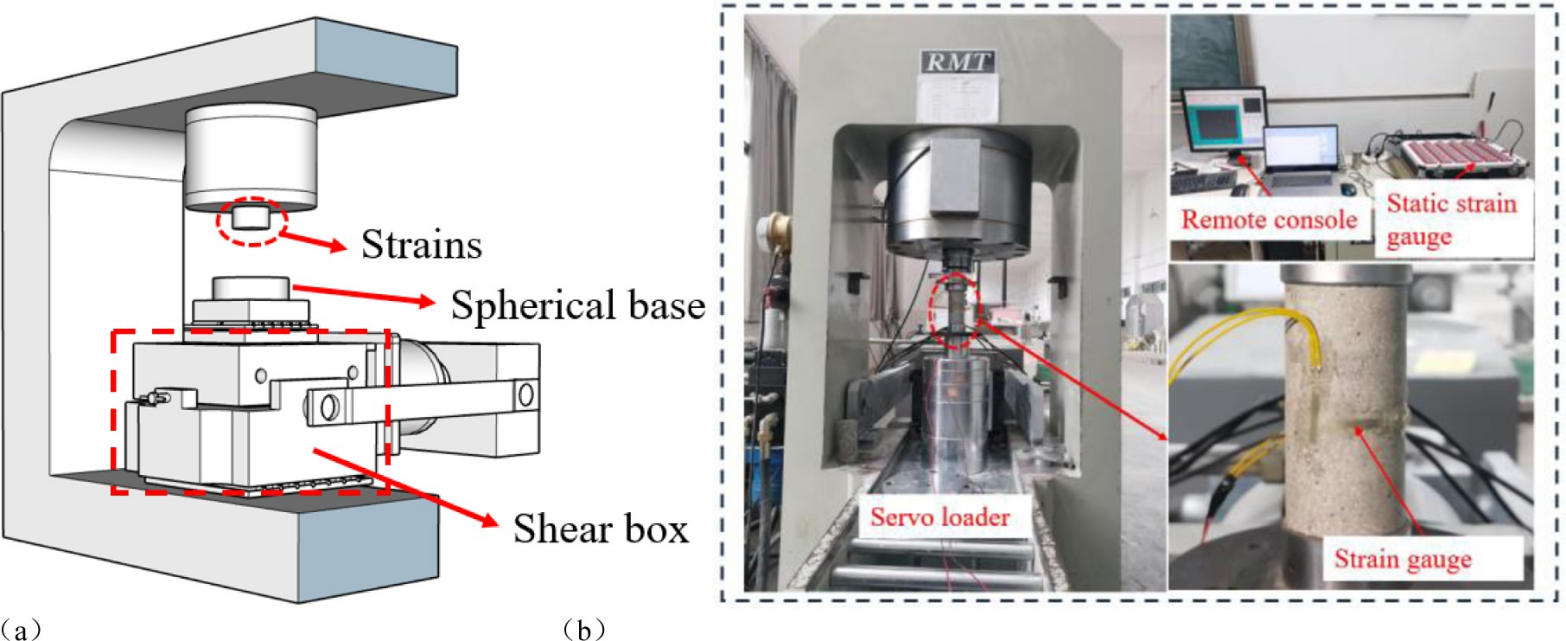
Presents a schematic diagram of the RMT-301 loading meter and uniaxial compression test site.

Cylindrical specimens (φ = 50 mm, H = 100 mm) and Brazilian disc-shaped specimens (φ = 50 mm, T = 50 mm) were prepared for uniaxial, Brazilian, and straight shear tests on the RMT-301 Mechanical Testing System for Rock and Concrete. Additionally, specimens containing interrupted joints (200 mm × 200 mm × 200 mm) were prepared for the same tests. The maximum axial load of the system is 1500 kN, with a loading rate of 0.01 ~ 90 kN/s or 0.0001 ~ 1 mm/s. The maximum peripheral pressure is 50 MPa, with a loading rate of 0.001 ~ 1 MPa/s. The axial and lateral displacements may reach up to 50 mm. A schematic diagram of the RMT-301 loading instrument is presented in Fig 3(A).

During the uniaxial test, the specimen was compressed at a constant loading rate of 0.1 mm/min. The axial stress-axial strain curves of the specimens were plotted using the data recorded automatically by RMT-301. The modulus of elasticity of the specimens was obtained by linear fitting of the straight line segments on the specimen’s axial stress-axial strain curves in accordance with the provisions of ISRM. As illustrated in Fig 3(B), strain gauges were affixed to the cylindrical specimen, which were employed to quantify the axial and radial strains during the elastic deformation phase of the specimen, and the Poisson’s ratio of the specimen was determined. For numerical simulation of uniaxial tests, the loading rate was 0.2 mm/s.

**Fig 4:**
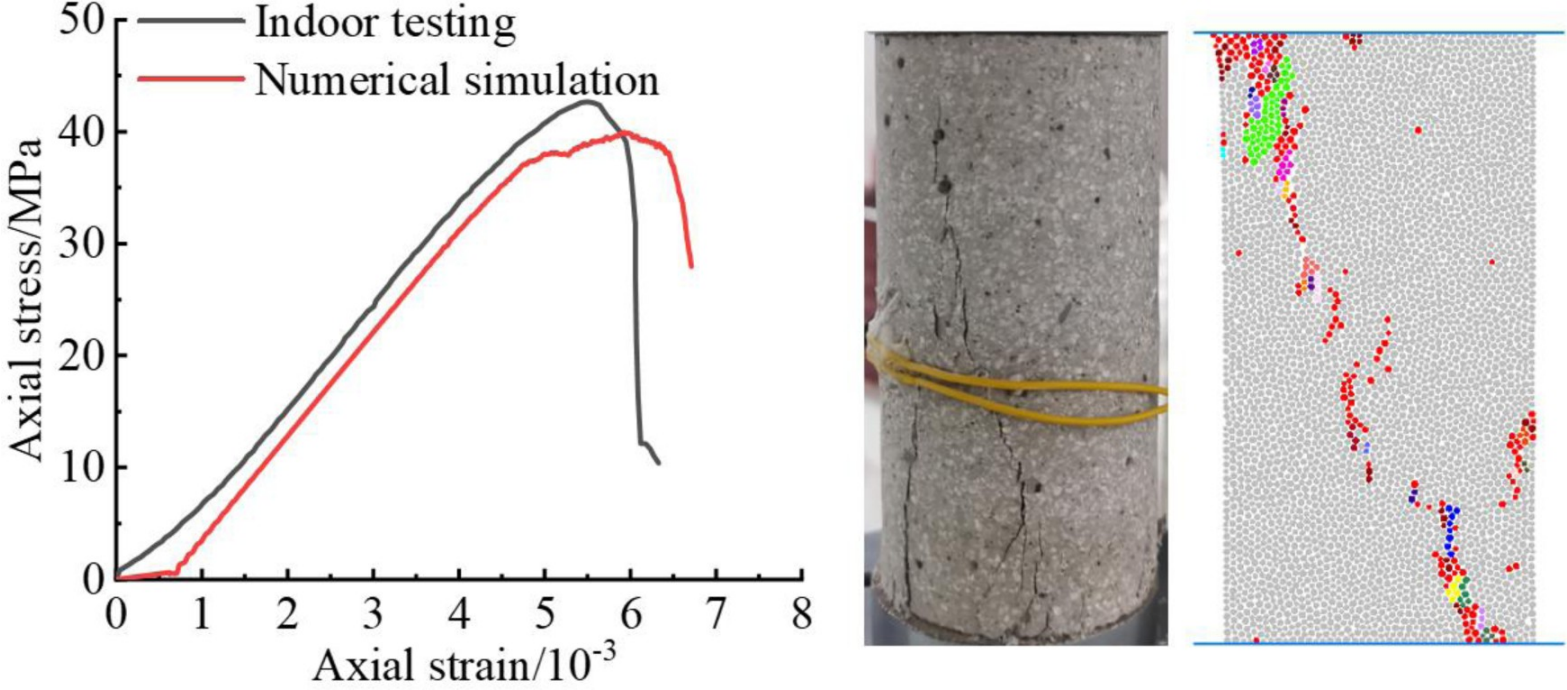
Presents a comparison of experimental and numerical results under uniaxial compression and damage modes.

**Fig 5:**
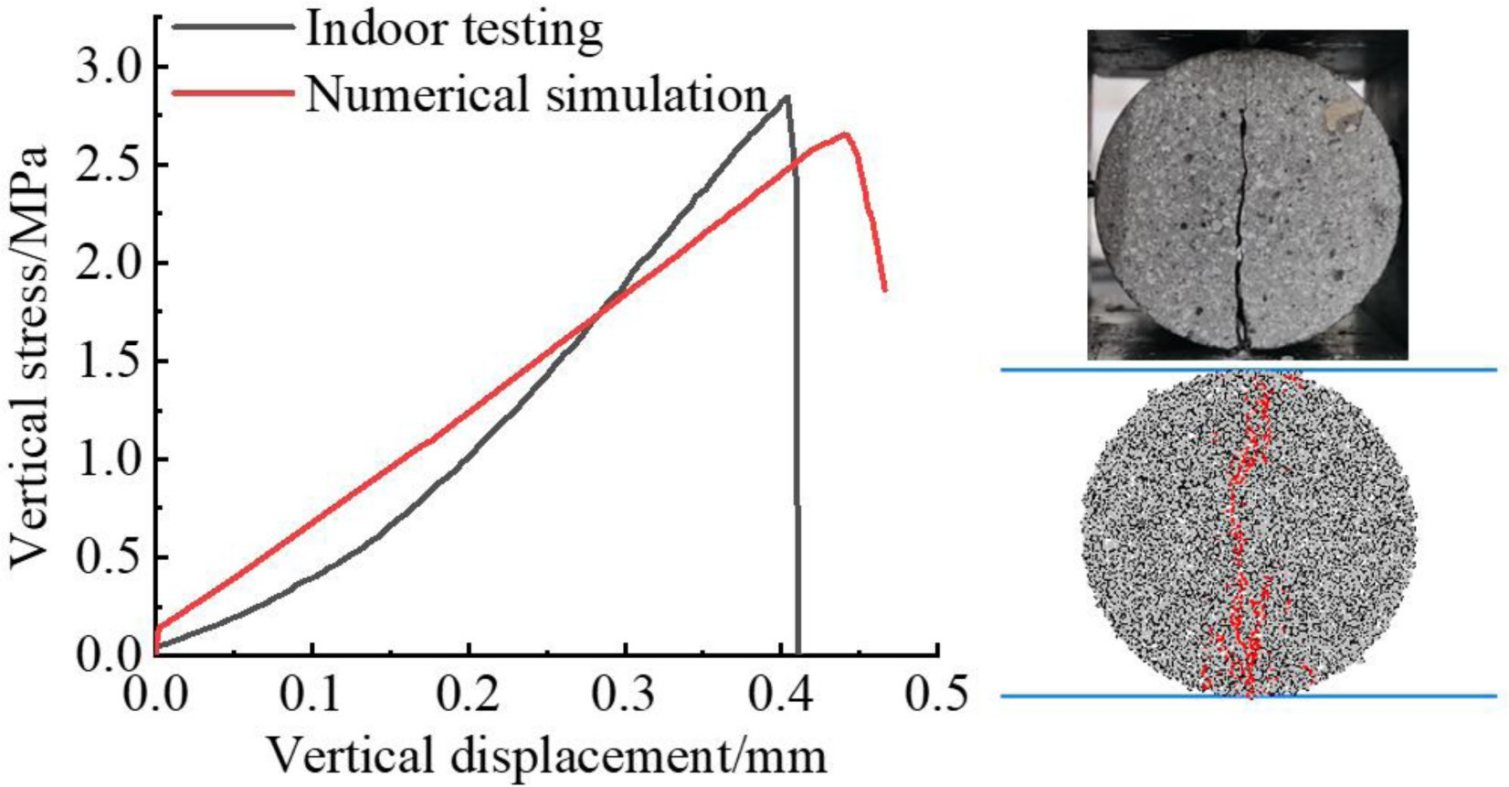
Comparison between Brazilian splitting tests and numerical results.

A series of "trial-and-error" experiments were conducted based on the rock-like material’s physico-mechanical parameters during the calibration of the fine-scale parameters of the numerical model. The resulting flow is shown in Fig 7(A). The simulation results are in general agreement with the experimental results, with the exception of discrepancies in the particle size (R), the normal and tangential stiffness of the particles (*k*_*n*_, *k*_*s*_), the normal and tangential stiffness of the bond ($\bar{k}n$, $\bar{k}s$), the normal and tangential strength of the bond ($\bar{\sigma }n$, $\bar{\sigma }s$), and so on. These discrepancies can be attributed to the fact that the simulation results were not adjusted to match the experimental results. The results of the uniaxial compression and Brazilian splitting experiments are presented in Figs 4 and 5, respectively.

A comparison of the mechanical parameters between the numerical simulation results and the test results is presented in Table 1. It can be observed that the simulated compressive strength and elasticity simulation under uniaxial test are in close agreement with the test data, with the deviations of the other parameters being within the acceptable range. The stress-strain curves all pass through the compression-density stage, the elasticity stage, the initiation and development stage, and the post-rupture stage. After the peak, the material exhibits a brittle fall nature, consistent with the general mode of uniaxial damage observed in conventional rocks. The damage modes observed are monoclinic shear damage. The simulated damage patterns of the specimens under the Brazilian splitting test are in good agreement with the test results. Consequently, the revised microparameters are capable of accurately representing the mechanical behaviour and deformation characteristics of rock-like materials.

**Table 1:** Presents a comparison of mechanical parameters between rock-like materials and those that have been numerically modelled.

	Uniaxial compressive strength (MPa)	Tensile strength (MPa)	modulus of elasticity (GPa)	Poisson’s ratio
**Experimental value**	**38.6**	**2.5**	**8.1**	**0.18**
**Simulated value**	**39.9**	**2.7**	**9.1**	**0.16**
**Relative error**	**3.4%**	**8.0%**	**12.3%**	**11%**

**Fig 6:**
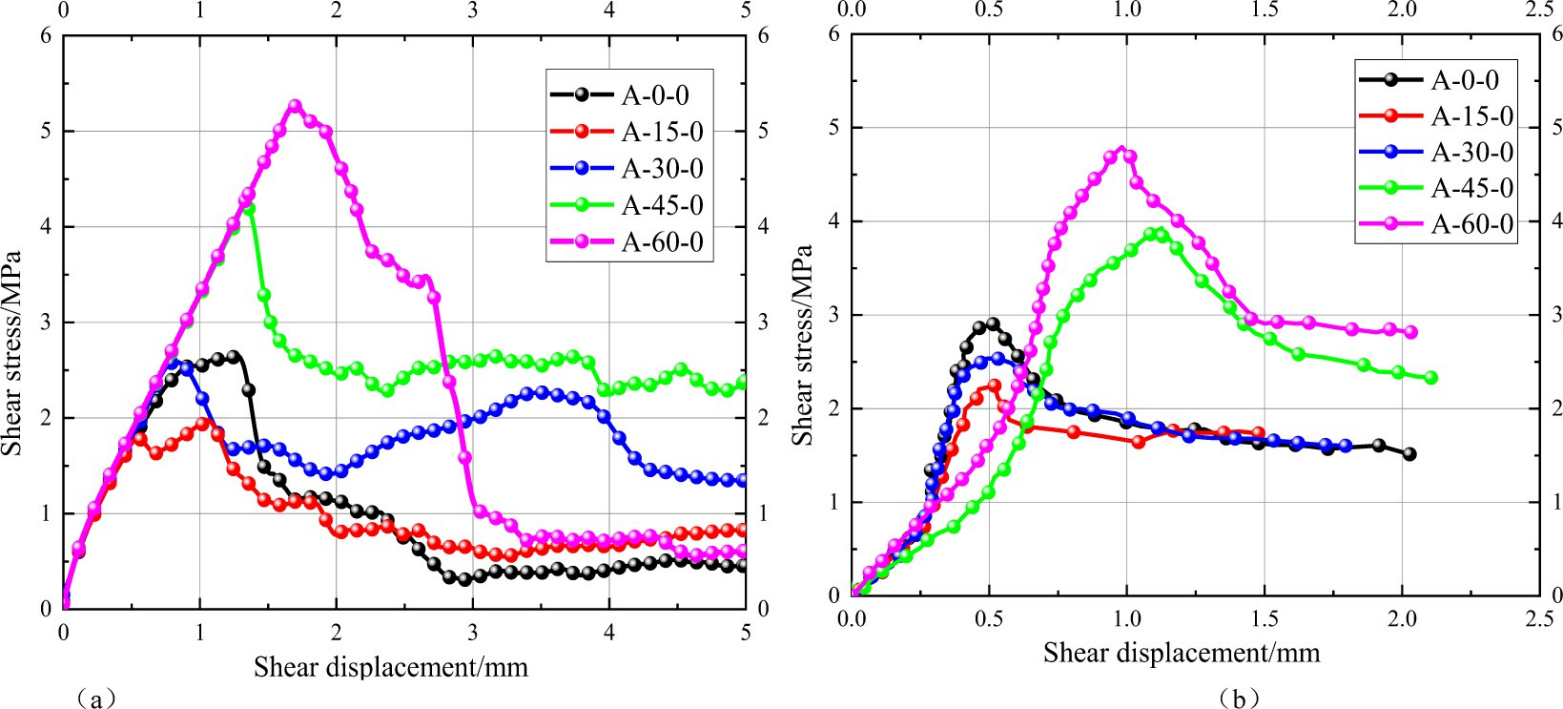
Numerical simulation and comparison of experimental stress-strain curves at different joint inclinations.

**Fig 7:**
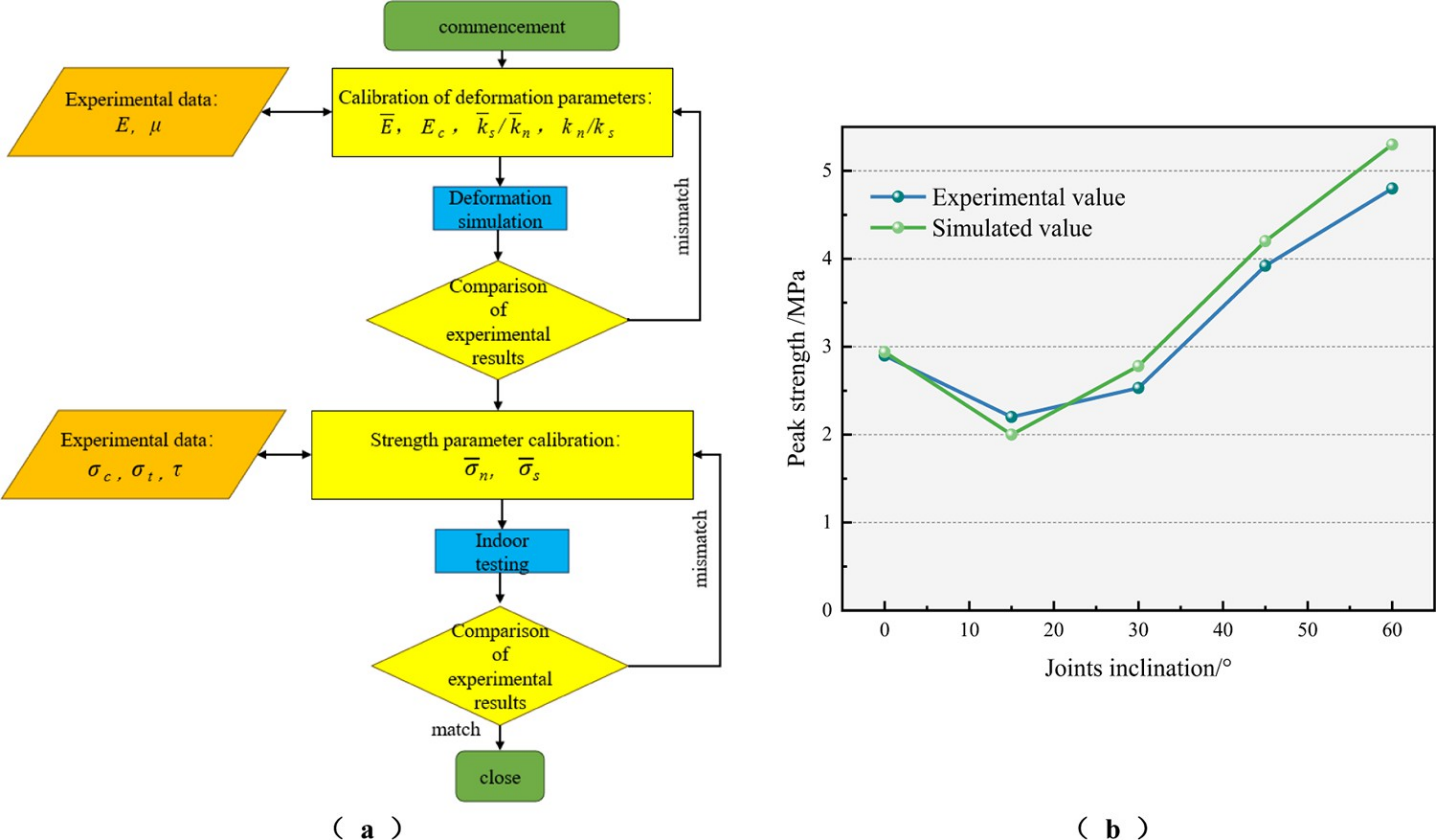
Parameter calibration process and comparison of simulation results with indoor test results. (a) Calibration process, (b) Peak shear strength.

Furthermore, in order to verify the reliability of the model, the simulated stress-displacement curves and the test stress-displacement curves of specimens with different joint inclinations are presented in Fig 6. It can be observed that the joint inclination exerts a significant impact on the shear mechanical behaviour of the test specimens and the numerical model. The simulation results presented in Fig 7(B) demonstrate a consistent trend with the test results, indicating a decrease in peak shear strength with an increase in joint inclination, followed by an increase. The minimum shear strength is observed at a joint inclination of 15°. It can be stated with a reasonable degree of certainty that the simulated and tested shear strength and damage mode aspects are essentially identical. However, there are some discrepancies between the simulated and experimental results. This may be attributed to the rigid nature of the particles in the PFC and the close contact between the particles, which may result in an inadequate representation of the effect of pre-existing defects in the rock mass in the numerical modelling. The corrected numerical model is more accurate in reflecting the shear mechanical behaviour of the actual specimen, as evidenced by the minimal discrepancy between the simulated and experimental values of the peak strength, which is less than 10%. The specific parameters utilized in the numerical model are presented in Table 2.

**Table 2:** Micro parameters determined in the simulation.

Particle parameters			Parallel bonding model parameters		
*E*_*c*_	Particle contact modulus (GPa)	4.3	$\bar{E}$	Parallel bonding modulus (GPa)	4.3
*R*_*min*_	Minimum particle size (mm)	0.087	${\bar{k}}_{s}$ / ${\bar{k}}_{n}$	Normal to tangential stiffness ratio	1.5
*R*_*max*_ */R*_*min*_	Particle size ratio	1.66	${\bar{\sigma }}_{n}$	Normal bond strength (MPa)	12
*ρ*	Particle density (kg*/m*^*-3*^)	2650	${\bar{\sigma }}_{s}$	Tangential bond strength (MPa)	12
*k*_*n*_*/k*_*s*_	Normal to tangential stiffness ratio	1.5			
*μ*	Coefficient of friction	0.2			

### 2.3 Numerical simulation programme

The simulation programme is divided into two groups, designated as A and B. Group A is for forward shear, while Group B is for reverse shear. The joint inclinations for forward shear are 0°, 15°, 30°, 45°, 60°, and 75°, while the joint inclinations for reverse shear are 90°, 105°, 120°, 135°, 150°, and 165°. The selected JRC values are 0, 5, 10, 15, and 20. The selected JRCs are 0, 5, 10, 15, and 20. A total of 36 working conditions are simulated, and A-α-β and B-α-β denote each working condition in Table 3. For example, A-30-5 denotes the numerical model with a joint inclination of 30° and a JRC of 5.

**Table 3:** Numerical simulation scheme.

Specimen	α/°	β	Specimen	α/°	β
**A-0-0**	**0**	**0**	**B-90-0**	**90**	**0**
**A-0-5**	**0**	**5**	**B-90-5**	**90**	**5**
**A-0-10**	**0**	**10**	**B-90-10**	**90**	**10**
**A-0-15**	**0**	**15**	**B-90-15**	**90**	**15**
**A-0-20**	**0**	**20**	**B-90-20**	**90**	**20**
**A-15-0**	**15**	**0**	**B-105-0**	**105**	**0**
**A-30-0**	**30**	**0**	**B-120-0**	**120**	**0**
**A-30-5**	**30**	**5**	**B-120-5**	**120**	**5**
**A-30-10**	**30**	**10**	**B-120-10**	**120**	**10**
**A-30-15**	**30**	**15**	**B-120-15**	**120**	**15**
**A-30-20**	**30**	**20**	**B-120-20**	**120**	**20**
**A-45-0**	**45**	**0**	**B-135-0**	**135**	**0**
**A-60-0**	**60**	**0**	**B-150-0**	**150**	**0**
**A-60-5**	**60**	**5**	**B-150-5**	**150**	**5**
**A-60-10**	**60**	**10**	**B-150-10**	**150**	**10**
**A-60-15**	**60**	**15**	**B-150-15**	**150**	**15**
**A-60-20**	**60**	**20**	**B-150-20**	**150**	**20**
**A-75-0**	**75**	**0**	**B-165-0**	**165**	**0**

## 3. Analysis of direct shear test results

### 3.1. Morphology of the shear stress-shear displacement curve

**Fig 8:**
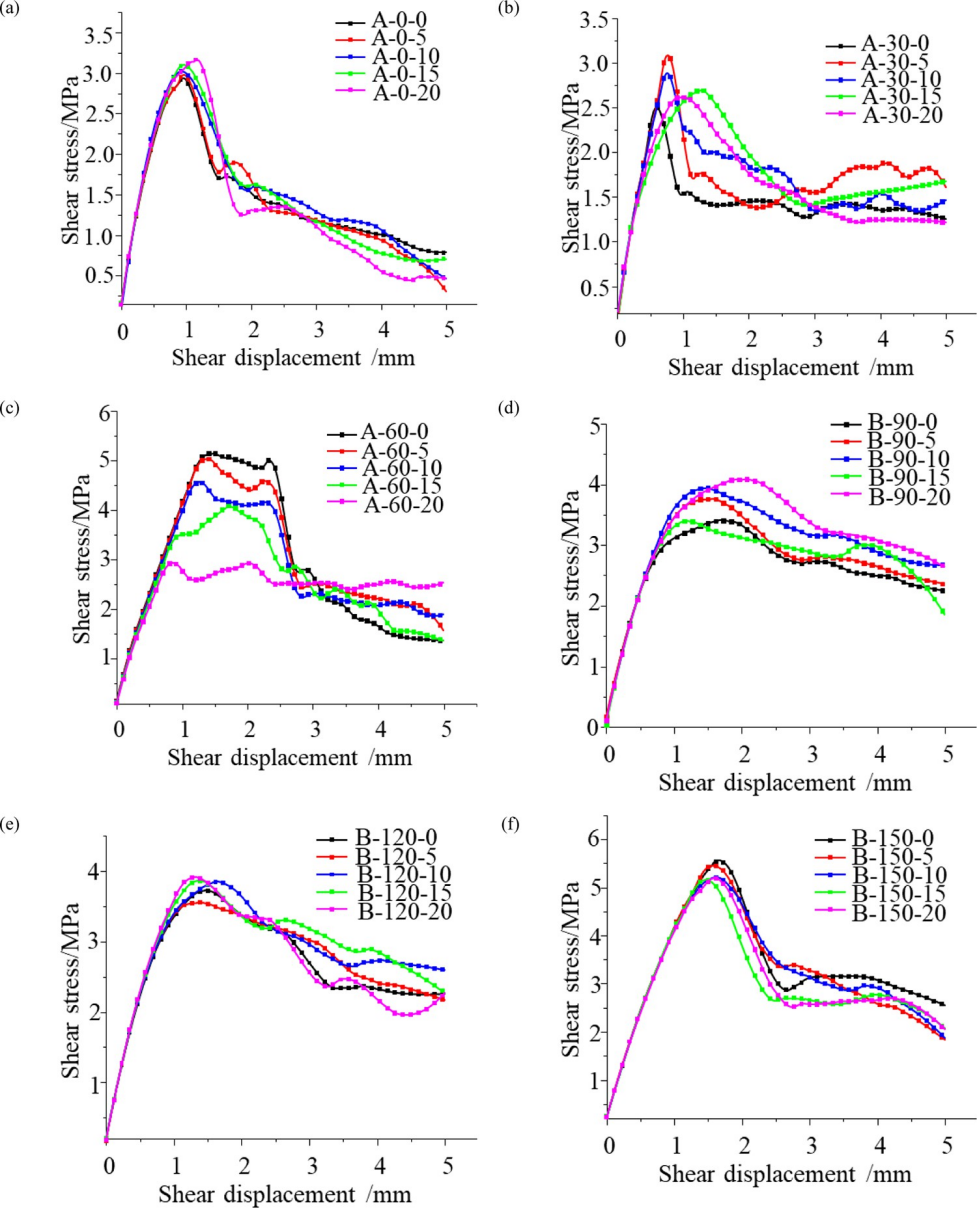
Shear stress-shear displacement curves for different roughness samples of the numerical model with joints (a) α = 0°, (b) α = 30°, (c) α = 60°, (d) α = 90°, (e) α = 120° and (f) α = 150°.

The modeled shear stress-shear displacement curves for different joint inclinations (0°, 30°, 60°, 90°, 120°, 150°) and joint roughnesses (0, 5, 10, 15, 20) are shown in Fig 8. There are obvious segmentation characteristics in the shear damage process of the model. Under normal stress, as the shear stress increases, the first stage is the elastic deformation stage where the shear stress increases rapidly with the shear displacement. The second stage is the stage of crack initiation and propagation at the tips of interrupted joints when the growth rate of shear stress becomes slower. The third phase is the rock-bridge damage penetration phase. The post-peak stress drops rapidly. The fourth stage is residual friction, where the rock bridge is damaged and penetrated and frictional occlusion from normal stress action resists shear stress.

The shear stress-shear displacement curves for conditions with the same joint inclination and different joint roughness have a high degree of similarity, and the elastic deformation stage curves almost overlap, but the peak stresses and residual strengths are different. The shear stress-shear displacement curve falls from the peak stress to the residual stress, the shear displacement travel is greater and falls less when the angle between the joint and the horizontal axis is greater, while the shear displacement travel is smaller and falls more when the angle between the joint and the horizontal axis is smaller (the fall is greatest at α = 0° and smallest at α = 90°). The larger the angle, the slower the post-peak stress decline, indicating that crack initiation and propagation of the non-coplanar continuation joints have better ductility at larger joint inclinations and that the destruction of the rock bridge did not result in a large decrease in the shear strength of the specimen. When the angle between the joint and the horizontal axis is small, the shear stress decreases substantially and sharply after the peak stress. This indicates that brittle damage is occurring in the specimen at this point.

The large difference in the shear stress-shear displacement curves for conditions with the same joint roughness and different joint inclinations is due to the qualitative change in the damage penetration path of the jointed rock mass as a result of the change in joint inclination. The degree of influence and the effect of changes in the roughness of the joint on the peak shear strength of the model varies with different inclinations of the joint. The effect of joint roughness on shear strength is small when the angle between the joint inclination and the shear direction is small (α = 0°, α = 150°) and large when the angle between the joint and the horizontal is large (α = 30°, α = 60°, α = 90°, α = 120°). In particular, the difference between the maximum peak stress and the minimum peak stress is greatest when the joint inclination is 60° and the joint roughness is varied.

### 3.2. Peak shear strength

**Fig 9:**
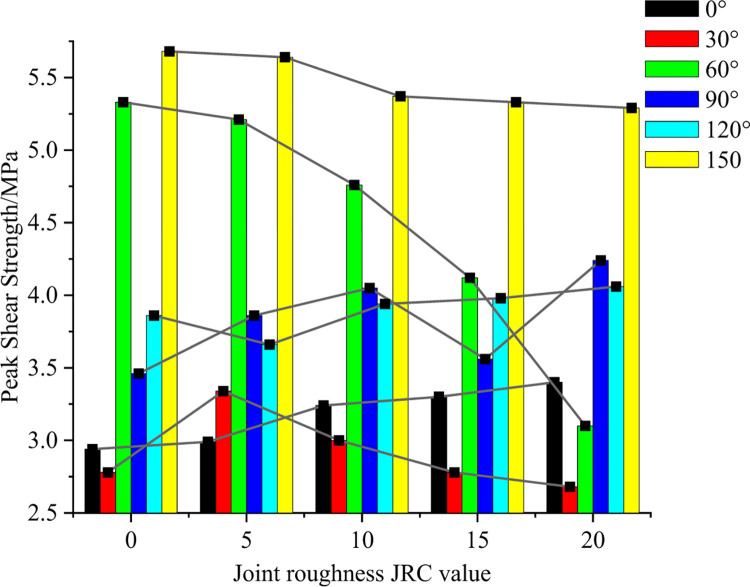
Variation in peak shear strength of specimens with different roughnesses.

The variation in peak shear strength of the numerical model containing joints of different roughness is shown in Fig 9. When the joint inclination is 0°, the peak shear strength increases gradually with a decreasing trend as the JRC value increases. When the joint is inclined at 30°, the peak shear strength increases and then decreases as the JRC value increases. For a joint inclination of 60°, the peak shear strength decreases with increasing JRC values and by an increasing amount. Except for a JRC value of 15, the peak shear strength increases with increasing JRC value when the joint inclination is 90°. Except for a JRC value of 5, the peak shear strength increases with increasing JRC value when the joint inclination is 120°. For a joint inclination of 150°, the peak shear strength gradually decreases as the JRC value increases.

The results show that both the inclination of the joint and the roughness of the joint affects the mechanical properties of the numerical model and that the effect of the inclination of the joint on the shear strength of the model is significantly greater than that of the roughness of the joint. The peak shear strength in the reverse shear direction was overall greater than in the forward shear direction, with the minimum peak shear stress at α = 15° and the maximum peak shear stress at α = 150°.

### 3.3. Analysis of changes in shear strength

In conjunction with the damage path diagram of cracks, this study explores the mechanism by which JRC values influence peak strength at different joint inclinations.

The effect of roughness on shear strength varies depending on the inclination, with a distinct impact observed at different angles. This paper posits that the effect of joint roughness on shear strength can be attributed to two factors: the friction effect and the damage effect. The evolution of these two effects is identified as the root cause of the increase in shear strength observed during the climbing damage mode and the decrease in the gnawing damage mode, which is attributed to the increase in roughness. The term ’friction effect’ is used to describe the process of crack extension through the formation of a tooth-shaped section. This occurs as a result of the shear process of the structural surface slipping along the slope of the force side of the tooth-shaped bump. Additionally, the increase in joint roughness causes the pressure slope to become rougher, leading to an increase in friction occlusion. This prevents the specimen from climbing and slipping, while also exhibiting stronger shear characteristics. The damage effect can be defined as the increase in joint surface with increasing joint roughness, which induces a greater number of microcracks during the shear process.

Under the action of forward shear, when the joint inclination angle is 0°, with the increase of JRC, the specimen must ‘climb’ and ‘gnaw off’ the tip of the joint bulge before the destruction of the process becomes more and more difficult, so the shear strength becomes greater. At a joint inclination of 30°, the crack extends to form a serrated penetration surface. JRC = 0, the sample damage is mainly a ‘climbing’ effect, JRC = 5, 10, 15, 20, the sample damage is mainly a ‘gnawing’ effect. As the JRC value increases from 0 to 5, the roughness of the joint increases, preventing the specimen from creeping and slipping and exhibiting stronger shear properties, thus increasing the shear strength. As the JRC value increases from 5 to 20, the degree of damage to the rock bridge increases, causing the shear strength to decrease. It is difficult for shear slip when the joint inclination angle is 60° because the specimen is mainly gnawed and the damaged penetration surface is more wavy. Increasing the JRC values did not alter the damage pattern. On the contrary, it further weakened the mechanical properties of the rock bridge, resulting in a gradual decrease in shear strength.

Under reverse shear, the crack propagation paths were altered at joint inclinations of 90° and 120°, and the initial cracks propagated towards the loading end rather than towards the inter-joint rock bridge, and the inter-joint rock bridge was not damaged. The weakening of the mechanical properties of the rock bridge due to the increase in JRC did not significantly affect the peak shear strength of the specimens due to the different penetration modes. At a joint inclination of 150°, the initial cracks expand outwards, secondary cracks agglomerate in the rock-bridge region, and increased JRC leads to more intense microcrack agglomeration in the rock-bridge region, reducing the peak shear strength.

After changing the shear direction from forward to reverse, the expansion penetration mode of the cracks changes, the initial cracks do not expand to the rock bridge area inside the joints, and the integrity of the rock bridge is better preserved, so the reverse shear strength is higher than the forward shear. At α = 15°, two initial cracks sprouted from the adjacent joint tips and expanded towards the rock bridge region, resulting in severe damage to the rock bridge. The joint tips were relatively close to each other, and the cracks developed and expanded rapidly to form a toothed through-path and undulated less, which made it easy for shear-slip damage to occur, and thus the peak shear strength was lower. At α = 150°, the initial crack extends outwards from the joint tip in the direction of the joint inclination, and the rock bridge between the two long cracks remains intact, allowing Ren to continue to carry the load, so the peak shear strength is higher.

## 4. Destruction processes and penetration patterns

It has been shown that the applied load leads to stress concentration at the tip of the joint and microcracks appear when the crack condition is reached. The number of induced microcracks gradually increases as the applied load increases. The mechanical properties of jointed rock are closely related to the processes of cracking, propagating, and fusing within the rock body. Therefore, the study of the cracking process of non-jointed specimens under shear loading can help to further understand the mechanical properties of jointed rock bodies.

### 4.1. Shearing in the forward direction

The number of cracks in the specimen subjected to forward shear is illustrated as a function of shear stress and shear displacement. The letters a, b, c, and d represent the microcrack evolution patterns of the joint rock model corresponding to the marked points. The figure shows grains in blue, joints in white and nascent cracks in red. Because of space limitation, only the part of the joint region is intercepted in the crack hair extension path diagram.

**Fig 10:**
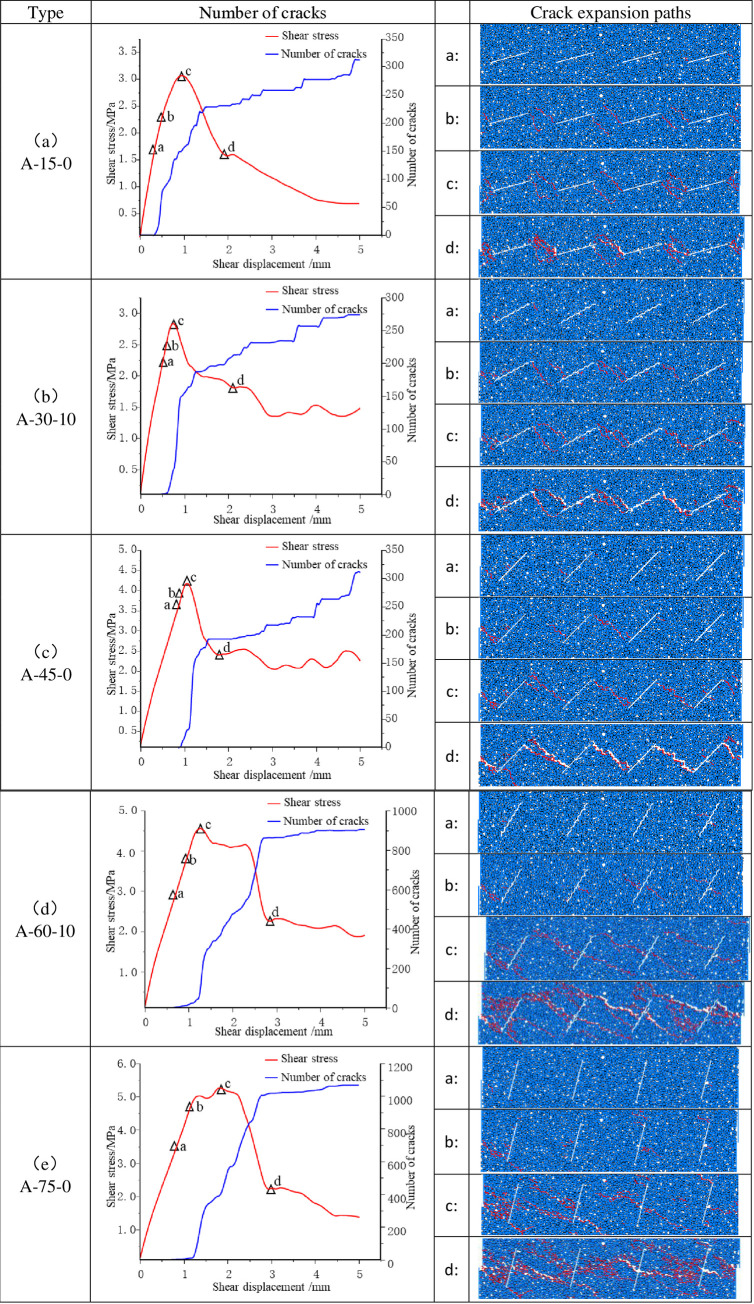
Number of cracks in the specimen under forward shear and the crack extension paths.

The evolution of the crack in the specimen with an angle of α = 15° and JRC = 0 is illustrated in Fig 10(A). With the increase in shear stress, microcracks emerged at the tip of joint No. 4, reaching point a. At point B, the crack progresses in a linear fashion towards the rock bridge, forming two parallel fissures. The minor depression of the joints results in the formation of cracks, which tend to coalesce at the tips of the rock bridges. At the peak stress at point C, the crack propagates to the neighbouring joints, namely the first and last lap, which adopt a toothed section. This phenomenon may be described as a ’climbing’ effect, which is particularly pronounced.

The crack extension path of the α = 30°, JRC = 10 specimen is illustrated in Fig 10(B). The crack initiates at the tip of the No. 1 joint and subsequently propagates in a vertical direction, ultimately converging with a neighbouring joint. At point C, the joints are characterised by a jagged appearance at the head and tail, accompanied by an increased level of roughness and a pronounced ’gnawing’ effect.

The crack extension of the α = 45°, JRC = 0 specimen differs from the first two conditions, as illustrated in Fig 10(C). At the point of peak stress (point C), the initial single crack in the rock bridge is of considerable significance. The section displays undulating but smooth characteristics, and the damage caused by shear slip is predominantly characterised by creep.

As illustrated in Fig 10(D), when α is equal to 60° and JRC is equal to 10, the nascent cracks emerge and propagate from the central region of the joint. At the peak stress point (c), the neighbouring joints are connected at both the head and the belly. The rough surface leads to a significant nibbling break.

The substantial inclination effect is observed at α = 75°, JRC = 0. As illustrated in Fig 10(E), multiple cracks emerge at point a, and the cracks at point b exhibit outward extension. As stress levels increase, secondary cracks emerge from the midpoint of the joints and converge with neighbouring joints at point C, resulting in an irregular section. The substantial decline in dip angle results in a prevalence of "nibble breaks," a multitude of microcracks within the bridge at point d, and the emergence of macro-shear crack zones through jointed zones.

As the joint inclination angle increased from 15° to 75°, the location of crack initiation shifted from the joint tip to the middle, and the expansion pattern changed from stable parallel cracks to complex irregular cracks. The JRC has a considerable impact on the damage pattern, with an increase in roughness at JRC = 10, which results in an intensification of the ’nibbling’ effect and an escalation of peeling wear following damage. The damage observed in the specimen under a small inclination angle can be primarily attributed to a ’climbing’ effect. However, with an increase in the inclination angle, a ’gnawing’ effect gradually becomes the dominant mechanism.

### 4.2. Shearing in reverse direction

**Fig 11:**
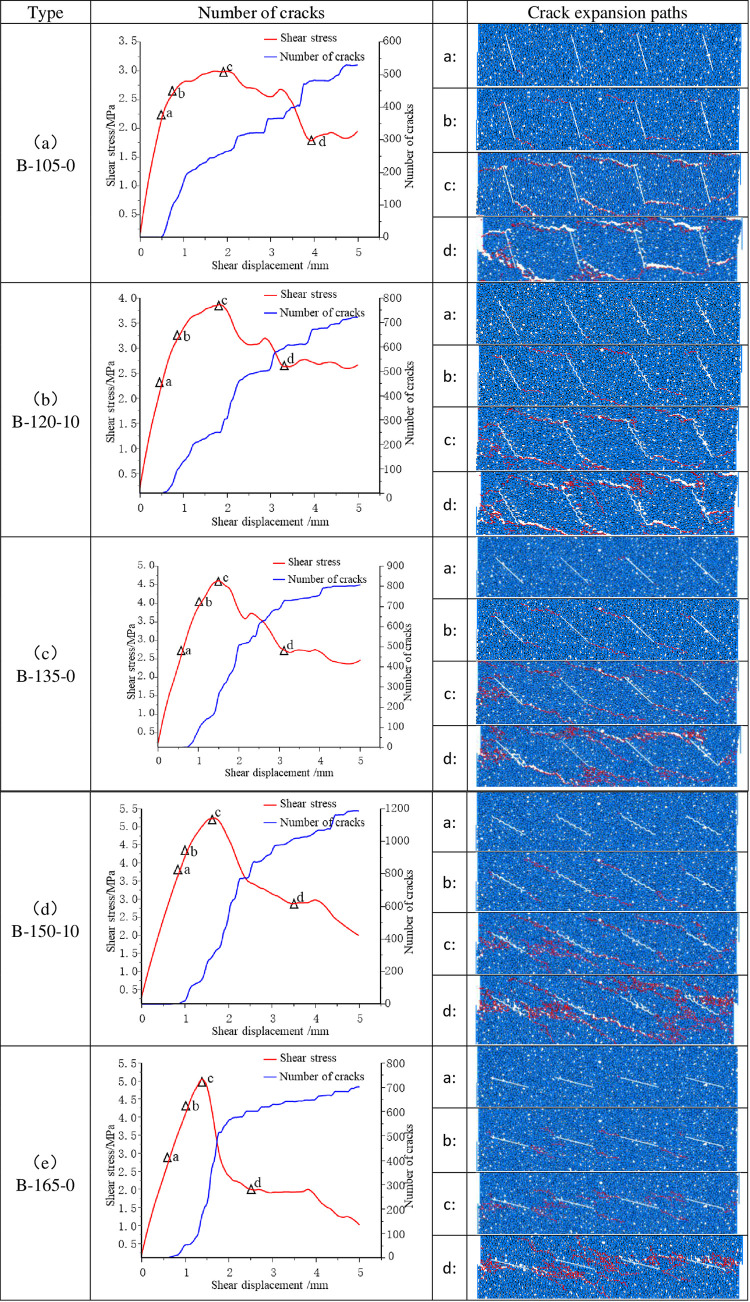
Number of cracks and crack extension path of the specimen under reverse shear.

As illustrated in Fig 11, alterations in the direction of shear result in corresponding modifications to the extension-penetration characteristics of the crack. When α equals 105° and JRC is equal to 0, as illustrated in Fig 11(A). The microcracks originated from the joint tip and subsequently expanded in the loading direction, ultimately converging with the adjacent joint tips to form the damage penetration pattern known as ’ first phase connected, last end connected ’. The rock bridge remains intact following the peak, with the generation of upper and lower macroscopic crack bands. The crack process of the specimen is analogous to that observed in the specimen sample at an inclination angle of 105°, with an angle of α = 120° and a JRC = 10.

At an angle of α = 135° and a JRC value of 0, the specimen’s rupture process is illustrated in Fig 11(C), which depicts the emergence and propagation of microcracks from the joint tips. The secondary cracks merge with the initial cracks, and the overall damage process exhibits some similarity with α = 105° and α = 120°.

As the joint inclination continued to increase, a new phenomenon emerged: initial crack expansion along the prefabricated joint direction. This phenomenon is illustrated in Fig 11(D). The formation of microcracked joints at the end of the joints was observed, accompanied by the emergence of secondary cracks at the tips of the rock bridges and joints. These cracks continued to propagate under continued loading, resulting in the formation of irregular penetration surfaces at the peak. This ultimately led to the destruction of the specimens, which demonstrated a distinct destruction mechanism from that observed in the previous example.

As illustrated in Fig 11(E), at α = 165° and JRC = 0, the crack extension is once more aligned with the joint inclination. This results in the formation of numerous secondary microcracks in the Rockbridge region, caused by the combination of a high inclination angle and the proximity of the joint tip. At the zenith of the phenomenon, the fissures converge entirely within the rock bridge, thereby forming a shear zone along the joints.

As the inclination of the joint increased from 105° to 165°, the pattern of crack initiation and extension gradually shifted from perpendicular to along the joint direction. This reflects the dominant role of inclination on crack extension. The impact of alterations in JRC (joint roughness coefficient) (0 vs. 10) on the crack extension pattern was found to be insignificant at smaller inclinations (e.g., 105°, 135°). However, at larger inclinations (e.g., 150°, 165°), an increase in JRC led to the emergence of secondary cracks in the rock-bridge region and a notable acceleration in the deterioration of the specimen.

### 4.3. Analysis of damage process and penetration patterns

**Fig 12:**
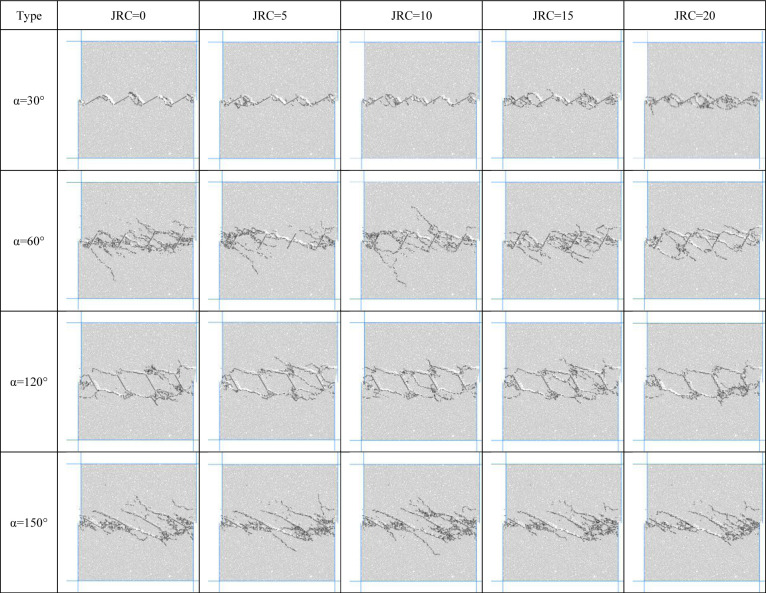
Destruction penetration patterns.

The joint inclination and joint roughness of the specimens under shear loading have an important influence on the crack process, and the crack distribution after shear damage of the specimens is shown in Fig 12. After the specimen reaches the crack initiation stress, microcracks sprout and expand steadily, and continue to produce a large number of secondary cracks, aggregation, and the formation of damage through the section, the stress reaches the peak value. From Figs 10 and 11, it can be concluded that, firstly, cracks sprout from the loading end under forward shear, and microcracks sprout from away from the loading end under reverse shear, and microcracks generally sprout from the tip of joints or the belly of joints. Secondly, the joint inclination angle determines the penetration mode, and the initial crack under forward shear extends along the direction of nearly vertical joints, forming a toothed penetration section with the first and last joints, and the damage of ‘creep-gnawing crack’ occurs. Reverse shear cracks expand along the direction of loading, forming two shear bands with the first phase and tail connected, and shear slip damage occurs. Finally, under forward shear, the slope and roughness together determine the destructive effect of the gear section; the rougher the joint, the greater the slope, the greater the ‘nibbling’ effect. Joint roughness has a large effect on shear strength as the initial crack propagates towards the rock bridge and an increase in JRC weakens the mechanical properties of the rock bridge.

**Fig 13:**
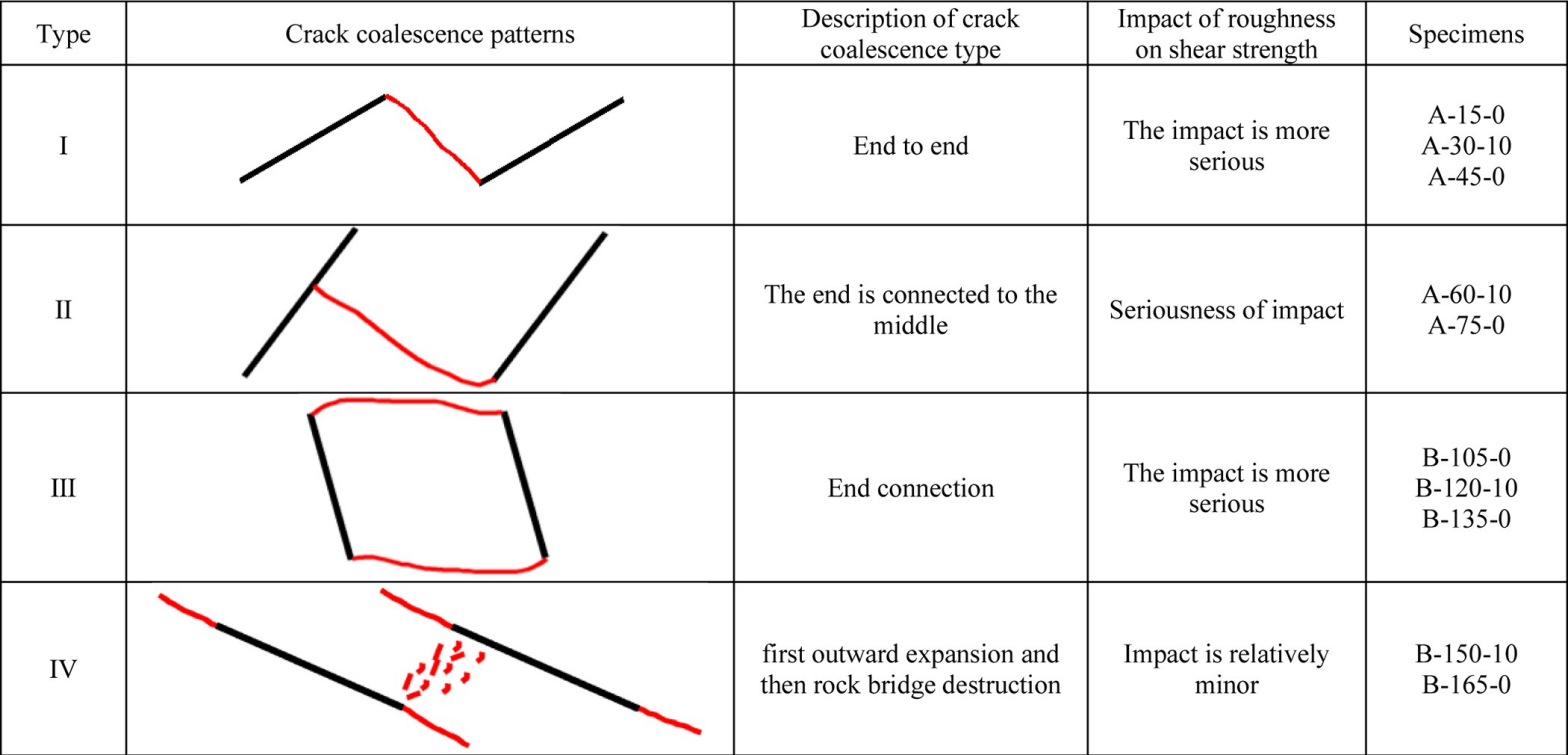
Types of crack consolidation.

The types of crack consolidation between joints are shown in Fig 13. When α = 15°, α = 30° and α = 45°, the penetration pattern of the sample is Type I. When α = 60° and α = 75°, the penetration pattern of the specimen changes to type II. When α = 105°, α = 120°, α = 135°, the penetration pattern of the specimen is Type III. When α = 150, α = 165°, the damage penetration pattern is Type IV. The inclination of the joint will determine the damage penetration pattern of the joint model and therefore, to a large extent, the shear strength. The degree of influence of joint roughness on shear strength varies at different inclinations.

## 5. Conclusion

This paper presents the findings of a simulation of a non-through rough four-joint straight shear test conducted under constant normal stress. The objective was to investigate the influence of joint roughness and joint inclination on the morphology of the specimen shear stress curve, peak shear strength, and damage mode. Additionally, the process and type of inter-joint crack merging during shear were analysed. The specific conclusions are as follows:

(1) It is demonstrated that the inclination angle of joints has a significant impact on the shear strength characteristics of jointed rock bodies. Furthermore, four penetration modes under different inclination angles are summarised. It is demonstrated that the effect of joint roughness on shear strength varies with inclination angle. This is not only reflected in the degree of influence, but also in the underlying mechanism and the resulting effect.

(2) At α = 0°, the peak shear strength gradually increases as the JRC value increases. At α = 30°, the peak shear strength increases and then decreases as the JRC value increases. At α = 60°, the peak shear strength decreases as the JRC value increases. At α = 90°, the peak shear strength increases with increasing JRC values, except for JRC = 15. At α = 120°, the peak shear strength increases with increasing JRC values, except for JRC = 5. At α = 150°, the peak shear strength decreases gradually with increasing JRC values.

(3) In order to ascertain the effect of shear directionality, a comprehensive comparison was conducted between the notable discrepancies in crack development pathways during forward and reverse shear. In the context of forward shear, the cracks propagate in a vertical direction within the joints, resulting in the formation of a tooth-like penetration surface. In contrast, the crack extension path during reverse shear is more complex, commencing at the nodal tip and extending towards the loading end, ultimately encircling the rock bridge.

(4) The influence of joint roughness on shear strength can be ascribed to the combined effects of friction and damage. The evolution of these two effects represents the fundamental cause of the observed increase in shear strength during the climbing damage mode and the concomitant decrease in the gnawing mode, which can be attributed to the increase in roughness.

## Supporting information

S1 Dataset Minimum data set. (ZIP)
